# Type 2 diabetes mellitus and sepsis: state of the art, certainties and missing evidence

**DOI:** 10.1007/s00592-021-01728-4

**Published:** 2021-05-10

**Authors:** Elisa Costantini, Massimiliano Carlin, Massimo Porta, Maria Felice Brizzi

**Affiliations:** 1 Department of Medical Sciences, University of Turin, Corso Dogliotti 14, 10126 Turin, Italy; 2 Azienda Ospedaliera Universitaria Città Della Salute E Della Scienza, Turin, Italy

**Keywords:** Type 2 diabetes mellitus, Sepsis, Immune dysfunction, Glycemic control, Organ dysfunction, Mortality

## Abstract

Diabetes and sepsis are important causes of morbidity and mortality worldwide, and diabetic patients represent the largest population experiencing post-sepsis complications and rising mortality. Dysregulated immune pathways commonly found in both sepsis and diabetes contribute to worsen the host response in diabetic patients with sepsis. The impact of diabetes on mortality from sepsis is still controversial. Whereas a substantial proportion of severe infections can be attributed to poor glycemic control, treatment with insulin, metformin and thiazolidinediones may be associated with lower incidence and mortality for sepsis. It has been suggested that chronic exposure to high glucose might enhance immune adaptation, leading to reduced mortality rate in septic diabetic patients. On the other hand, higher risk of acute kidney injury has been extensively documented and a suggested lower risk of acute respiratory distress syndrome has been recently questioned. Additional investigations are ongoing to confirm the protective role of some anti-diabetic treatments, the occurrence of acute organ dysfunction, and the risk/benefit of less stringent glycemic control in diabetic patients experiencing sepsis. Based on a MEDLINE/PubMed search from inception to December 31, 2020, the aim of this review is therefore to summarize the strengths and weaknesses of current knowledge on the interplay between diabetes and sepsis.

## Introduction

Sepsis is defined as a “*life-threatening organ dysfunction caused by dysregulated host response to an infection”*, and represents a leading cause of death worldwide, with a mortality rate > 10% [1]. In 2017, almost 50 million incident cases of sepsis were estimated worldwide and 11 million sepsis-related deaths were reported, representing nearly 20% of all global deaths [2]. Septic shock is highly prevalent in the general population, occurring in the 8–10% of Intensive Care Unit (ICU) patients, with a high mortality rate (almost 40%) [3]. The expanding elderly population suffering from extensive comorbidity burden, physiological frailty and immune senescence [4] leads to predict an increased mortality rate for sepsis over the next couple of decades [5].

With the rising globalization of Western diet and lifestyle, the incidence of Type 2 Diabetes Mellitus (T2D) is increasing and its prevalence is expected to exceed 700 million worldwide in the near future, reaching pandemic proportions [6]. T2D and diabetes-related complications are also a leading cause of hospitalization, disability and mortality [7, 8].

Although still under debate [9–11], several lines of evidence indicate that diabetic patients have an increased risk of infection [9, 12–17], and a 2 to 6 times higher risk of sepsis compared to the age-matched non-diabetic people [12, 17]), and higher sepsis-related morbidity and mortality compared to non-diabetic individuals [12, 15, 18, 19]. Diabetic patients are also likely to have higher rates of colonization by resistant pathogens, including methicillin-resistant Staphylococcus aureus, than non-diabetics [20]. These considerations support the finding that diabetes is an increasingly common comorbidity among septic patients [21, 22]. As a matter of fact, during a 25-year study period (1979–2003), sepsis occurred in 12.5 million of 930 million acute-care hospitalizations, and diabetes was reported in 17% of cases [21]. Moreover, diabetic patients account for the largest population experiencing post-sepsis complications and rising mortality [15].

Despite current improvements in diagnosis and treatment options, diabetes and sepsis remain common, costly and lethal worldwide [3, 11, 14]. This work aims at reviewing the current state of knowledge about: (1) the impact of diabetes and sepsis on the immune system, (2) the influence of diabetes on the risk of sepsis and its outcomes, and (3) the optimal target for blood glucose control during sepsis in patients with diabetes.

A MEDLINE/PubMed search was conducted from inception to December 31, 2020, using the MeSH terms *Diabetes mellitus* AND *Sepsis* AND the following: *Immune system processes, Glycated hemoglobin, Insulin, Hypoglycemic agents, Metformin, Sulphonylurea compounds, Thiazolidinediones, Incretin, Multiple-organ dysfunction syndrome, Lung injury, Acute respiratory distress syndrome, Acute kidney failure, Blood glucose, Mortality.*

All types of publications and articles related to human studies were initially included. Out of 583 records retrieved through the initial database search, 425 remained after removing duplicates. After manual assessment based on title/abstract, 150 remained for full-text assessment for eligibility. Articles without full text or not written in English, case reports and studies involving generically critically ill patients or patients with specific infective focus were excluded. Based on these exclusion criteria, 92 records were excluded, while 58 articles remained. An additional 46 records were identified by manual search among the references cited in these records and further assessed for eligibility according to the above-mentioned criteria, leading to exclude 27 and include 19.

**Fig. 1:**
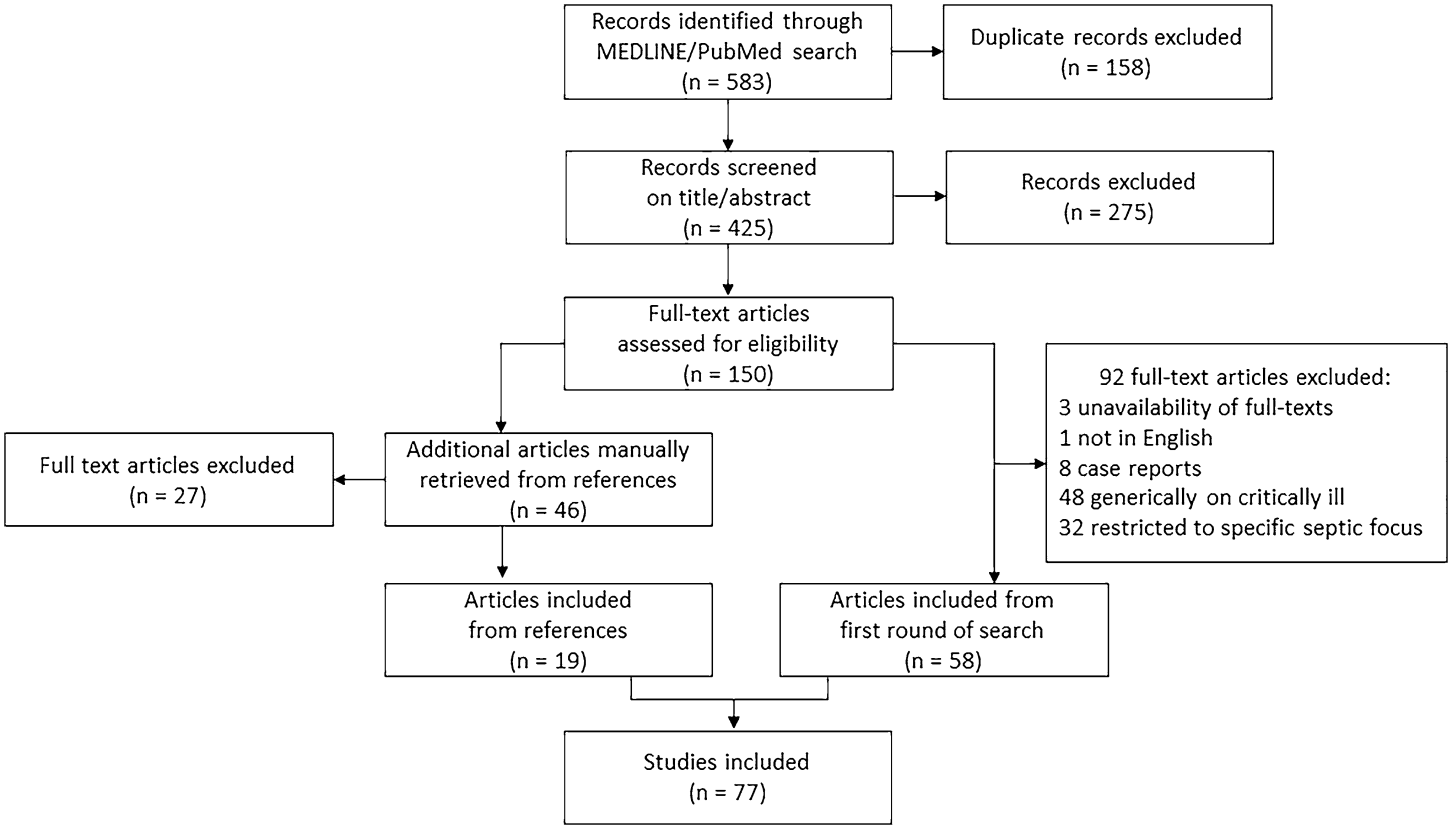
Study flow diagram

Finally, 77 studies were included in the qualitative analysis (Fig. 1).

## Interactions between type 2 diabetes and sepsis

T2D is a complex clinical syndrome characterized by persistent hyperglycemia, associated with decreased insulin secretion and sensitivity [13]. Several metabolic abnormalities, including inflammation and insulin resistance driven by both chronic and stress-induced hyperglycemia, and T2D-related obesity and dyslipidemia, additionally worsen the host response against infections.

Also, sepsis exerts a global impact on the immune system, impairing the lifespan, generation and function of innate and adaptive immune cells and leading to perturbation of the immune homeostasis [23].

**Fig. 2:**
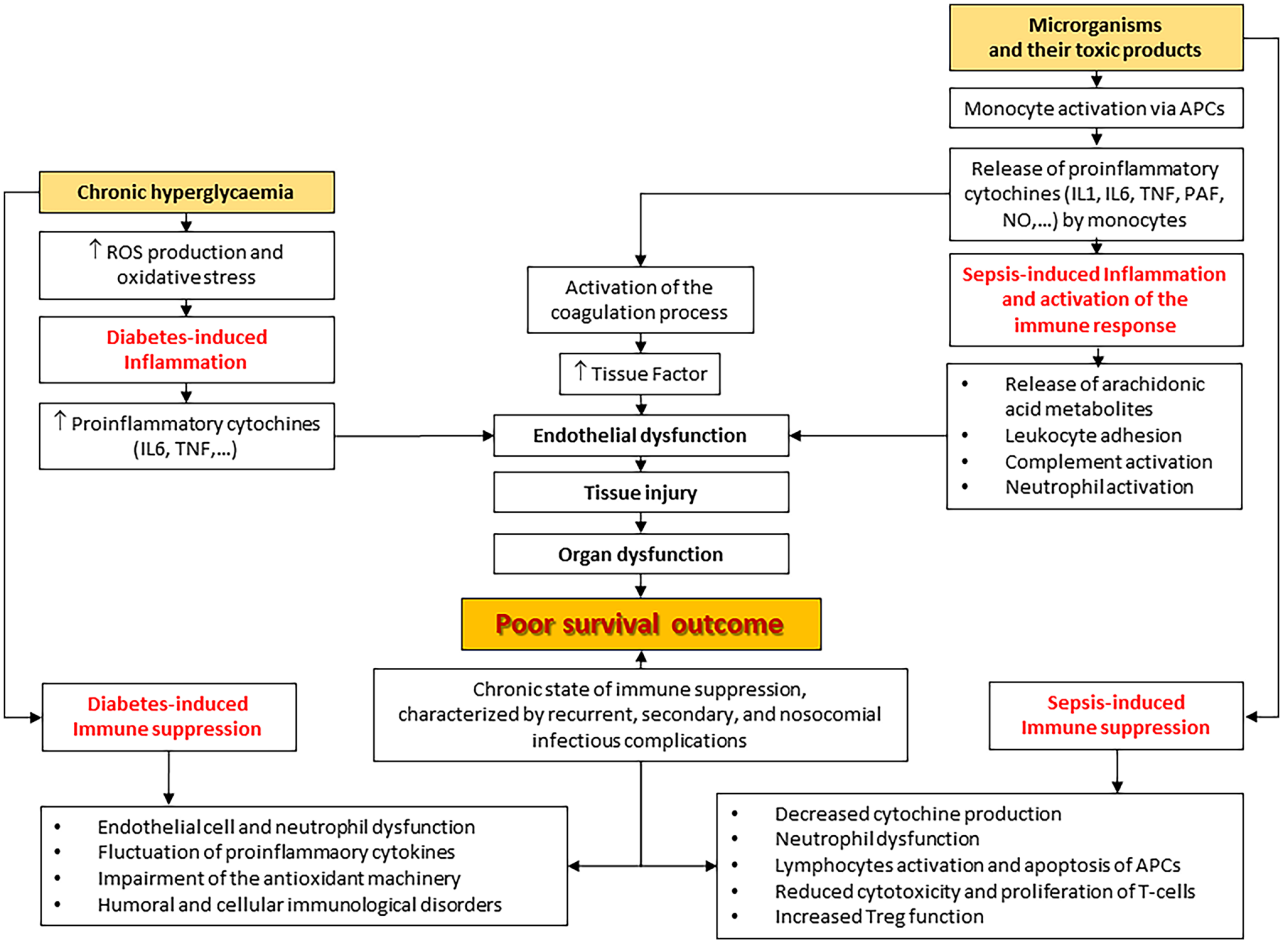
Interactions between diabetes and sepsis in inflammation and on the immune system ( adapted from Tiwari et al. [13]). Both T2D-related chronic hyperglycemia and toxic products released by invading microorganisms during sepsis contribute to increase inflammatory response [13]. It is generally accepted that the chronic and indolent inflammation induced by T2D and obesity differs from the acute inflammatory response caused by sepsis [77]. However, Frydrych et al. [15] outlined the impairment of several inflammatory responses in both T2D and sepsis (data not shown in the Figure), including: **a** increased levels of complement proteins (which are defective in T2D) driving systemic inflammation, organ failure and mortality; **b** mitochondrial dysfunction and redox imbalance as relevant mediators of disease progression; **c** impaired calcium homeostasis promoting elevated inflammatory responses, cellular dysfunction and toxicity. The increase in pro-inflammatory cytokines, induced by both T2D and sepsis, and the activation of the immune system due to sepsis are responsible for the endothelial dysfunction carrying the organ dysfunction characteristic of sepsis and accountable for poor outcome [13]. Additionally, functional neutrophil defects and deranged recruitment into sites of infection are commonly found not only in T2D but also in sepsis. Apoptosis of both lymphocytes and antigen-presenting cells (APCs) is a hallmark of septic-mediated immune suppression, whereas endothelial cell dysfunction, fluctuation of pro-inflammatory cytokines and the impairment of both the antioxidant machinery and humoral immunity are linked to TD2 [15]. Thus, in diabetic patients surviving from sepsis, the coexistence of the sepsis-induced immune activation over-described and such immune suppression related to both T2D and sepsis weakens the immune response contributing to create a chronic immune suppression leading to further infective complications and poor long-term survival [15]

Currently, the molecular network that cooperates to worsen clinical outcomes in patients with T2D and sepsis remains uncertain [15]. Figure 2 summarizes the current knowledge on the mechanisms of sepsis and the effects of chronic hyperglycemia, both impacting on the immune system and translating into poor patient outcome [13, 15].

## Premorbid modifiers of the risk of sepsis

### Long-term glycemic control and the risk of sepsis

Glycated hemoglobin (HbA1c), term used to describe “*a series of stable minor hemoglobin components formed slowly and nonenzymatically from hemoglobin and glucose*”, is the most widely used marker of long-term glucoregulation and represents a risk mark for the development of diabetes complications [11, 24]. In hyperglycemic sepsis, it allows to distinguish non-diabetic individuals experiencing stress hyperglycemia from patients with previously undiagnosed diabetes and, comparing actual blood glucose values with the HbA1c-estimated average levels at preadmission, to identify stress-induced glycemic deterioration in patients with preadmission diagnosis of diabetes [25].

Only a few studies have investigated the relationship between glycemic control and infectious diseases [26, 27]. A recent review of higher-quality population-based epidemiological studies [26] have reported an association between high HbA1c (> 7–8% or > 53–64 mmol/mol) and a 1.5–3.5-fold increased risk of infection in diabetic patients. However, these studies are still debated, since their statistical power and controls for confounders are missed.

A further large-size retrospective cohort study [27] on more than 150,000 patients, among whom approximately 85,000 were diabetics (mostly T2D), confirmed a powerful association between poor glycemic control and high risks of serious infections (not just sepsis). Specifically, diabetic patients showed greater hospitalization risks for infections compared to non-diabetics, regardless of glycemic control (sepsis rates were elevated even among patients with HbA1c < 6% or 42 mmol/mol). Nevertheless, for several infections, an association trend was found between increasing HbA1c level and the risk of infection. Within diabetic patients, a poor metabolic control was associated with a threefold risk of hospitalization. Overall, 15.7% of infection-related deaths, 16.5% of infection-related hospitalizations, 6.8% of infections requiring a prescription, and up to 20% of sepsis cases, have been attributed to HbA1c value different from 6–7% (42–53 mmol/mol). In detail, the incidence rate ratio for sepsis ranged from 1.2 (for HbA1c ≥ 7% or 53 mmol/mol) to 3.64 (for HbA1c ≥ 11% or 97 mmol/mol). Interestingly, even a tight metabolic control (HbA1c < 6% or 42 mmol/mol) was associated with an increased risk of infections in the older population, among whom the infectious risk and poor outcomes were found globally higher. The authors hypothesized for these patients that a less stringent glycemic control (up to HbA1c 8%; 64 mmol/mol) may be beneficial, while a tighter control would be associated with additional risks [27].

Thus, although evidence suggests that a better glycemic control might reduce the risk of infections, further trials including older patients, people with a poor metabolic control, and whit a history of significant infectious disease are required [26, 27].

### Impact of insulin and other anti-diabetic medications on the incidence and mortality for sepsis

Immunomodulatory effects of both insulin and non-insulin glucose-lowering agents have been extensively documented, and their beneficial impact in diabetic patients with sepsis has been suggested [9, 11, 28–32].

Insulin may protect against over-activation of the immune system by preventing the adverse effects on immune functions related to high blood glucose and exerting direct and indirect anti-inflammatory effects [9, 28]. However, two large-size observational studies failed to reveal differences in mortality attributable to previous insulin treatment. A first report on critically ill subjects (among whom 7% with previously diagnosed insulin-treated diabetes) [33] revealed that formerly insulin-treated diabetic individuals were more severely ill, however, they did not display an increased mortality rate. In a further prospective observational study [34], including ICU septic patients with and without diabetes (the first either insulin- and non-insulin treated), the disease progression and mortality for sepsis in diabetic patients was similar regardless of insulin treatment.

Some non-insulin glucose-lowering agents have been associated with several immune-modulating effects in preclinical studies. Specifically, metformin may exert important pleiotropic effects, involving the regulation of lactate metabolism and AMPK activation, and produce anti-inflammatory, anti-endotoxemic, vasoactive and antimicrobial actions [31]. Thiazolidinediones (TZD) increase neutrophil migration, suggesting potential benefits in the modulation of the inflammatory response and in the outcome of septic patients [11]. An anti-inflammatory action has been shown and an immunomodulatory effect has been hypothesized also for incretin hormones, since they are involved in inflammatory response [30, 32, 35]. Preclinical models of sepsis have demonstrated that incretin-based therapies decrease immune cell activation, inhibit pro-inflammatory cytokine release and reduce organ dysfunction and mortality [32]. Although incretin-based therapies have not yet been tested in clinical trials of sepsis, it has been hypothesized that both incretin-mimetics [32] and DPP4 inhibitors [35] may exert positive pleiotropic effects on both inflammation and immunomodulation. On the contrary, insulin secretagogue-mediated off-target effects driven by the inhibition of the adenosine triphosphate-sensitive potassium channel in β cells were found to impair the immune response against invading pathogens in preclinical studies [29].

A large nested case–control study analyzing the impact of current treatment with non-insulin agents on the incidence of sepsis [29] demonstrated that metformin may confer a persistent benefit on the rate of hospitalization for sepsis. TZD administration was also inversely associated with the occurrence of sepsis, unlike meglitinide. Treatment with sulfonylureas and DPP4 inhibitors is not associated with altered incidence of sepsis. In a recent meta-analysis, also Liang et al. [31] linked metformin treatment with reduced mortality in diabetic patients with sepsis. Nevertheless, although of interest, the reliability of this observation is limited by the relatively small sample size. A more recent, larger population-based cohort study [36] reported that metformin treatment is not significantly associated with the risk for sepsis nor with 30-day mortality for sepsis in diabetic patients. Although some small clinical trials in critically ill patients have suggested potential benefits in glycemic control using incretin infusion, these studies included mixed populations and had limited power [32].

Thus, an association between preadmission treatment with insulin or non-insulin glucose-lowering agents and the risk and outcome of sepsis remains controversial. The degree of glycemic control, rather than the anti-diabetic therapies, could explain the risk and mortality for sepsis. As a matter of fact, in a small observational study [37], HbA1c has been proved an independent prognostic factor for hospital mortality and time of hospitalization for diabetic septic patients, while no difference in the outcomes were found related to prior anti-diabetic treatments.

Further, clinical trials specifically investigating the potential benefits of anti-diabetic medications in septic cohorts are required.

## Optimal blood glucose control during sepsis

Progression of sepsis is associated with changes in insulin and cortisol circulating levels, resulting in significant glucose perturbations, organ damage and activation of the immune system [38]. Besides the well-known stress-induced hyperglycemia, hypoglycemia may also reflects a pathological acute stress response. Indeed, hypoglycemia is commonly associated with sepsis and considered an epiphenomenon of severe organ dysfunction preceding death. Although the mechanisms and relationships between hypoglycemia and the severity of the disease in septic patients are still debated, the role of inflammatory cytokines has been proposed [39].

In critical settings, derangement of glycemic control is associated with more severe disease and poorer prognosis [39–41]. However, diabetes may modulate the relationship between dysglycemia and mortality in sepsis [40]. Indeed, the risk of mortality associated with hyperglycemia is lower in diabetic than non-diabetic patients [42] and is not influenced by hypoglycemia [39] or glycemic variability [43].

Despite strong recommendations for early insulin administration, how to monitor and treat stress-induced hyperglycemia remains under debate [41, 44].

Several large-size trials have investigated the optimal acute blood glucose control in critically ill patients, including septic ones [22, 45–47]. However, only a few small studies were restricted to septic patients [48, 49], and none specifically targeted diabetic patients. Table 1 reports the main clinical trials evaluating the impact of different targets of acute glycemic control in critically ill and septic patients. Van den Berghe et al. [45] first evaluated patients admitted to surgical Intensive Care Units (ICU) who were randomly assigned to receive intensive insulin therapy (blood glucose target 80–110 mg/dl) or conventional therapy (target 180–200 mg/dl). Although the number of septic patients was not reported at baseline, intensive insulin therapy reduced episodes of nosocomial septicaemia of about 46% and the proportion of patients requiring prolonged antibiotic therapy. Specifically, a tight glucose control (TGC, i.e., blood glucose levels < 110 mg/dl) was associated with lower morbidity and mortality rates (with a 43% relative risk reduction of ICU mortality). However, this result relies of the benefit obtained in the subgroup of those patients staying in ICU for more than 5 days and in cardiac surgical patients (accounting for the majority of the study population) who previously received intravenous glucose load for nutritional purpose. In a subsequent study in medical ICU patients, the same group [46] failed to confirm a benefit on mortality in the overall population, since demonstrated that TGC prevents morbidity in all patients, but reduces mortality only in those staying in the ICU for at least 3 days. Moreover, concerns raised on the high rates of hypoglycemic events (more than sixfold higher than the previous study) in this subgroup of patients. A post-hoc analysis [50] of pooled data from the two Leuven studies [45, 46] further confirmed that TGC carried a significantly higher risk of hypoglycemia (which occurred in 11.3% of patients on TGC vs. 1.8% of those on conventional insulin therapy, *p* < 0.0001). However, even if hypoglycemia was not associated with early deaths and/or neurological sequelae, a higher risk of death was reported. Such pooled data finally revealed that TGC significantly reduced morbidity and mortality in mixed medical/surgical ICU (particularly in patients staying in ICU at least 3 days). In addition it was reported that all patient subgroups, including those admitted for sepsis, benefit from TGC. Only for diabetic patients, no survival benefit was reported. A rapid normalization of blood glucose levels rather than hypoglycemic events has been proposed to explain the lack of TGC benefit in diabetic patients.

**Table 1:** Clinical trials evaluating the impact of different targets of acute glycemic control in critically ill and septic patients

Authors	Year	Patients	Study design	Main findings
Trials showing benefits on mortality in patients on tight glycemic control				
Van den Berghe et al. [45]	2001	1,548 surgical (63% cardiac) ICU patients receiving mechanical ventilation (the number of septic patients not reported; 204 diabetic patients)	Prospective, randomized, controlled trial	TGC versus 180–200 mg/dl: Lower overall ICU mortality (8.0% vs. 4.6%, *p* < 0.04) due to benefit in patients staying in the ICU for > 5 days (10.6% vs. 20.2%, *p* = 0.005) Greater reduction in mortality observed in septic patients with multiple-organ failure 34% lower overall in-hospital mortality No impact on mortality in diabetic patients
Trials showing benefits on mortality in patients on less stringent glycemic control				
Van den Berghe et al. [46]	2006	1,200 medical ICU patients considered in need of ICU for ≥ 3 days (total number of septic patients not reported, but sepsis reported as a major trigger for admission to ICU; 203 diabetic patients)	Pospective, randomized, controlled trial	TGC versus 180–200 mg/dl: No overall difference in mortality (37.3% vs. 40%, *p* = 0.33) In patients staying in the ICU for ≥ 3 days lower in-hospital mortality (reduction of in-hospital mortality from 52.5% to 43%, *p* = 0.009) but higher rate of hypoglycemic episodes No impact on mortality in diabetic patients Lower overall morbidity (prevention of acquired AKI, earlier weaning from mechanical ventilation, and earlier discharge from the medical ICU and the hospital—but no detectable reduction in bacteremia)
Brunkhorst et al. [48]	2008	537 patients with severe sepsis/septic shock (163 diabetic patients)	multicenter, randomized trial	TGC vs. 180–200 mg/dl: Higher rate of severe hypoglycemia (17.0% vs. 4.1%, *p* < 0.001) and serious adverse events (10.9% vs. 5.2%, *p* = 0.01) No difference in 28-day nor 90-day mortality No difference in survival between patients with and without diabetes
Preiser et al. [47]	2009	1,001 medico-surgical ICU patients (445 medical patients—number of septic patients not reported; 203 diabetic patients)	Prospective, randomized, multi-center, controlled trial (*Glucontrol* Study)	TGC versus 140–180 mg/dl: Higher rate of hypoglycemia (8.7% vs. 2.7%, *p* < 0.0001) No difference in ICU, in-hospital and 28-day mortality No association between mortality and diabetes
Finfer et al. [22]	2009	6,104 medico-surgical ICU patients considered in need for ICU for ≥ 3 days (1,302 patients with severe sepsis; 1,211 diabetic patients)	Large, international, randomized trial (NICE-SUGAR Trial)	TGC versus ≤ 180 mg/dl: Higher 90-day mortality (OR 1.14 [95% CI 1.0–1.3]) without differences among medical and surgical patients (OR respectively 1.3 and 1.1, *p* = 0.10) No difference in mortality between patients with and without diabetes Higher rate of severe hypoglycemia (6.8% vs. 0.5%, *p* < 0.001)

Citations are in order of publication date

ICU = Intensive Care Unit; TGC = Tight Glucose Control (blood glucose levels targeted to 80–110 mg/dl)

However, further studies failed to confirm these benefits from TGC [22, 46–48], although differences in study design, selection of patients, nutritional support, targeted glucose range and blood glucose measurements make the comparison challenging [41]. As a matter of fact, a further trial specifically involving patients with severe sepsis [48] not only failed to demonstrate a benefit on mortality from TGC, in both diabetic and non-diabetic patients, but was early stopped for safety reasons (e.g., a significantly increased rate of severe hypoglycemic events). Two further large-scale trials including mixed populations of medical and surgical patients, the *Glucontrol* study [47] and the *Normoglycemia in Intensive Care Evaluation—Survival Using Glucose Algorithm Regulation* (NICE-SUGAR) *Trial* [22], reported higher rates of hypoglycemia in the TGC group. The former, prematurely stopped for the high rate of unintended protocol violations, did not find differences in mortality from TGC, while the second one revealed that a less stringent glycemic control translates into lower mortality rate, regardless of diabetes. Finally, a recent meta-analysis by Yamada et al. [51] confirmed the absence of clinical benefits of a stringent glycemic control in term of mortality, while reporting an increased rate of hypoglycemia in both diabetic and non-diabetic patients on TGC compared to patients on mild (140–180 mg/dl) and very mild control (180–220 mg).

The U-shaped curve describing the relationship between glycemic control and mortality (patients with low and high glucose levels have worse outcomes than those in the normal/moderate range) suggests that moderately elevated glycemic level may represent the ideal target in diabetic patients [9, 15, 52]. However, whether this effect was actually due to such glucose levels or to confounding variables driving hypoglycemia and poor outcome is still a matter of debate [52]. Additionally, the relationship between longer *time in blood glucose range* 70 to 140 mg/dl and lower mortality rate, clearly described in non-diabetic patients, is missing in diabetics [53].

Current guidelines recommend to treat hyperglycemia in critical patients to a target of 140–180 mg/dL, regardless of the presence of previously known diabetes [54, 55]. The need for specific targets of glycemic control in diabetic patients has been postulated [44, 56, 57], and some studies have suggested that less stringent glycemic control (e.g., targeting blood glucose levels at 180–250 mg/dL) may be beneficial in critical patients with premorbid chronic hyperglycemia (e.g., HbA1c level > 7% or > 53 mmol/mmol). However, concerns (including increased risk of infection, glycosuria and polyneuropathy) have raised against such permissive glucose levels in critically ill diabetic patients [58]. Based on these observations, Egi et al. [58] proposed to adopt a uniform blood glucose target for patients with and without diabetes (≤ 180 mg/dL), at least until the randomized control LUCID trial (*Liberal GlUcose Control in Critically Ill Patients with Preexisting Type 2 Diabetes trial*) [59] will inform on the risks and benefits of more liberal glucose control strategies.

Finally, a role was suggested for closed-loop glucose control systems and immunomodulatory treatment options, to avoid hypoglycemia during insulin therapy and to control the rise in circulating cytokine levels in diabetic patients with severe sepsis and septic shock [60].

## Acute organ dysfunction during sepsis

The occurrence of organ dysfunction in diabetic patients with sepsis was first evaluated in a cohort of 12.5 million people admitted to hospital for sepsis between 1979 and 2003, among whom over 2 million had diabetes [21]*.* The study revealed that diabetic patients were less likely to develop acute respiratory failure (9% versus 14%, *p* < 0.05), regardless of the source of infection, but more likely to develop acute renal failure (13% versus 7%, *p* < 0.05) than non-diabetic ones. No differences were found in dysfunction of other organs (cardiovascular failure occurred in the 4% of the overall population, while hepatic, hematological, metabolic and central nervous system dysfunction globally occurred in the 6%).

In both sepsis and chronic hyperglycemia, injury of the glycocalyx, due to generation of reactive oxygen species and inflammatory mediators, impacts the microcirculation and leads to organ damage [61]. The coexistence of diabetes and severe sepsis additionally compromises red blood cell deformability, worsens the microcirculation and hastens the progression of organ dysfunction [62].

A recent retrospective observational study [61] supports the link between premorbid chronic hyperglycemia and progression to organ dysfunction in septic patients. The authors demonstrated that, in septic patients admitted to the ICU, HbA1c values ≥ 6.5% (≥ 47.5 mmol/mmol) were independently associated with the progression of liver and kidney failure within 72 h, and with ICU mortality. Only, a positive trend for the progression of lung and cardiac dysfunction and clotting disorders was reported.

Unlike lower risk of acute respiratory dysfunction [14], higher risk of acute kidney injury in diabetic patients was confirmed in a large nationwide retrospective study [63] and in a recent meta-analysis [14].

### Acute respiratory failure

Acute Lung Injury (ALI) and Acute Respiratory Distress Syndrome (ARDS) are common life-threatening conditions in critically ill patients. A protective role of diabetes against the development of ALI/ARDS has been extensively documented in different cohorts of critically ill and septic patients [28, 63–67]. However, such protection has recently been questioned, since a meta-analysis by Wang et al. [14] demonstrated a similar incidence of respiratory dysfunction in diabetic and non-diabetic septic patients.

Moreover, the potential mechanisms for such presumptive protective effect are still unclear [21, 28, 63, 66, 67]. Among the proposed mechanisms the impaired neutrophil function and altered neutrophil–endothelial interaction [68], the immunomodulatory effects mediated by hyperglycemia and resulting in a impaired response against endotoxin-mediated injury [28, 68], as well as the presence of obesity and dyslipidemia-related metabolic effects [28] are included. Finally, some potential protective effects may result from extensive and earlier medical care and the anti-inflammatory properties of anti-diabetic medications, including insulin, TZD and metformin [21]. Similarly, whether and how diabetes may conversely contribute to increased incidence of other organ dysfunction in sepsis, such as renal failure, is still unclear [66].

### Acute kidney injury (AKI)

AKI develops in one-fourth of all septic patients and half of those with bacteremia or shock [69]. In diabetic patients, observational studies reported an incidence of AKI ranging from 27% [63] to 73% [70].

Although diabetes is an established risk factor for both AKI [21, 33] and sepsis [9, 12–16], and reported as an independent risk factor for persistent renal dysfunction in ICU septic patients developing AKI [70], the impact of diabetes in increasing the risk of AKI is still debated.

As a matter of fact, in a prospective single-center ICU study [71], elevated serum creatinine on the first day was associated with the occurrence of AKI in patients with severe sepsis, though not in diabetic patients. In a further large cross-sectional multicenter study involving patients with severe sepsis or septic shock, diabetes resulted not associated with the occurrence of AKI or the need for renal replacement therapy [70]. Despite these findings and the wide heterogeneity of data related to the incidence of AKI in diabetic septic patients, the above-mentioned meta-analysis [14] confirmed that the incidence of AKI is definitely increased (over 50%) in diabetic compared to non-diabetic septic patients.

## Mortality from sepsis

Increased susceptibility to infection and sepsis in diabetes is extensively documented [9, 12–17], but equivocal results from epidemiological studies pose doubts on the association between diabetes and increased risk of infection-related morbidity and mortality [9–11]. Different study populations (including lack of stratification into Type 1 and Type 2 Diabetes, different adjustments for comorbidities, sepsis etiology and disease severity), drug administration regimens to control blood glucose and methods to measure outcomes have been proposed to explain this heterogeneity [10, 11].

Table 2 reports recent clinical studies investigating the association between diabetes and mortality for sepsis. Among them, a large-size observational study [72] demonstrated that diabetic patients experienced an increased mortality from infectious diseases (persisting even after adjustment for comorbidities) and a twofold increased risk of mortality for sepsis compared to the general population. Additionally, two large-size retrospective cohort studies [12, 19] found higher mortality rate related to infections in diabetic compared to non-diabetic patients, whereas others [33, 34, 63, 66, 70, 73, 74] failed to demonstrate such association, and Esper et al. [21] even reported improved survival in diabetic patients.

**Table 2:** Recent clinical studies investigating the association between diabetes and mortality for sepsis

Authors	Year	Patients	Study design	Main findings
Studies showing adverse association between diabetes and mortality for sepsis				
Zoppini et al. [72]	2018	185,341 diabetic individuals	Retrospective cohort study on a regional electronic archive	Increased risk of death from infection-related causes in diabetic people (especially in female and people aged between 30 and 64 years): Overall SMR 1.83 (95% CI, 1.71–1.94) SMR for septicaemia 1.91 (95% CI, 1.76–2.06)
Shah et al. [12]	2003	513,749 diabetic individuals (matched to an equal number of non-diabetics)	Retrospective cohort study on population-based administrative data	Higher global infection-related mortality in diabetic patients (including home and hospital) (risk ratio up to 1.92 [99% CI 1.79–2.05]) No significant difference in term of infection-related hospital mortality (risk ratio up to 0.94 [99% CI 0.87–1.01])
Bertoni et al. [19]	2001	9,208 individuals (533 with diabetes)	Retrospective cohort study on a national registry	Higher infection-related mortality in diabetic patients with cardiovascular disease (RR 3.0 [95% CI 1.8–5.0])
Studies showing no association between diabetes and mortality for sepsis				
Van Vught et al. [74]	2017	41,492 ICU septic patients (8085 with diabetes)	Retrospective large national database review	No association between diabetes and adjusted 90-day mortality In diabetic patients, only severe hypoglycemia in absence of hyperglycemia is associated with increased 90-day mortality (OR 2.95 [95% CI 1.19–7.32]), whereas in non-diabetics several combinations of abnormal glucose level were associated with increased 90-day mortality
Van Vught et al. [34]	2016	1,104 ICU septic patients (241 with diabetes)	Prospective observational study	No association between diabetes and 90-day mortality: HR 0.90 [95% CI 0.69, 1.15] after correction for BMI, age, gender, hypertension, cardiovascular and renal insufficiency HR 1.02 [95% CI 0.81–1.29] after correction for APACHE IV score
Venot et al. [70]	2015	10,911 patients (3,728 with severe sepsis or septic shock; among them, 451 with diabetes)	Case–control study based on a multicenter database	No difference in mortality between diabetic and non-diabetic septic patients (19.8% vs. 15% in the matched case–control analysis; *p* = 0.08)
Chang et al. [63]	2012	16,497 ICU septic patients (4,573 with diabetes)	Nationwide population-based retrospective cohort study	No association between diabetes and 90-day mortality (OR 0.972 [95% CI 0.890–1.061]) after adjustment for age, gender, comorbidities and number of acute organ dysfunction
Yang et al. [66]	2011	9,221 septic patients (2,943 with diabetes)	Retrospective large database review	No difference in-hospital mortality between diabetic and non-diabetic septic patients (19.2% vs. 20.0%; *p* = 0.37)
Stegenga et al. [73]	2010	830 ICU patients with septic shock (188 with diabetes)	Retrospective analysis of a previously published RCT	No difference in mortality between diabetic and non-diabetic septic patients: 28-day mortality 31.4% vs. 30.5%, *p* = 0.8235 90-day mortality 39.1% vs. 39.0%, *p* = 0.9827
Vincent et al. [33]	2010	3,147 ICU septic patients (226 with insulin-treated diabetes)	Prospective study	No difference in ICU and hospital mortality between diabetic and non-diabetic septic patients
Studies showing a protective effect of diabetes on mortality for sepsis				
Esper et al. [21]	2009	12,500,459 septic patients (2,070,459 with diabetes)	Retrospective large national registry review	Lower hospital mortality in diabetic vs. non-diabetic patients (18.5% vs. 20.6%, *p* < 0.05)

Citations are in descending order of publication date

SMR = Standardized Mortality Ratio – ICU = Intensive Care Unit – RCT = Randomized Controlled Study

Few observational studies have investigated the link between premorbid glycemic state and sepsis outcome, showing that HbA1c levels at admission are in direct correlation with hospital mortality in diabetic patients with sepsis [37, 61].

The results by Tayek et al. [75], firstly reporting a global benefit on mortality from diabetic status, were confirmed in the meta-analysis by Wang et al. [14], which demonstrated that diabetes is not associated with adverse outcomes in patients with sepsis, while beneficial. As a matter of fact, some studies notably demonstrated an association between hyperglycemia and increased mortality in non-diabetic patients, unlike in diabetic patients, suggesting that acute hyperglycemia may drive different pathophysiologic effects in diabetic and non-diabetic patients. Nevertheless, whether the link between hyperglycemia and mortality in non-diabetics relies on hyperglycemia-induced toxic effects or is the hallmark of disease severity still remains to be clarified [9]. Although the mechanisms for such protective effect driven by diabetes remain uncertain, previous exposure to high glucose has been proposed to enhance immune adaptation and to induce benefits [9, 14, 75]. The role of inflammation has been also investigated in this context. In particular, Stegenga et al. [73] reported comparable cytokine levels and procoagulant responses in critical septic patients with and without preexisting diabetes, while a different study unveiled the presence of elevated levels of markers of endothelial cell activation in patients with diabetes and septic shock, compared to patients without diabetes [76]. Beneficial effects of insulin administration, prevention of acute lung injury, adaptation to previous oxidant stress and nutritional intake in obese patients with diabetes were also proposed as protective against sepsis [9].

## Conclusions

Sepsis represents a rising cause of mortality worldwide and diabetes is a common and increasing comorbidity in septic patients. Although the higher risk of infection in diabetic patients is well documented, the impact of diabetes on the outcome of sepsis and the mechanisms underlying their interactions are still debated.

Critical issues that need clarifying include the impact of diabetes and sepsis on the immune system, the role of glycemic control and the potential protective role of anti-diabetic treatments, on the occurrence of sepsis and its outcome, including the risk of renal failure and acute respiratory dysfunction. Also, recommendations for glycemic targets during sepsis do not stand on firm grounds.

Further large-size prospective studies, randomized controlled trials whenever possible, specifically including diabetic patients with sepsis instead of generically critically ill or patients with specific infective focus, could clear some of these unsolved questions, including the risk/benefit balance of more liberal acute glycemic control.

Finally, interesting and challenging therapeutic options, including immunomodulatory approaches targeting the pathways activated in T2D and sepsis, are under investigation and may result in clinical benefits.
